# Elevated ambient temperature reduces fat storage through the FoxO-mediated insulin signaling pathway

**DOI:** 10.1371/journal.pone.0317971

**Published:** 2025-02-26

**Authors:** Tucker Hopkins, Cole Ragsdale, Jin Seo

**Affiliations:** Department of Biology, College of Arts and Sciences, Rogers State University, Claremore, Oklahoma, United States of America; The University of Alabama at Birmingham Heersink School of Medicine, UNITED STATES OF AMERICA

## Abstract

Temperature profoundly impacts all living organisms, influencing development, growth, longevity, and metabolism. Specifically, when adult flies are exposed to high temperatures, there is a notable reduction in their body fat content. We investigate the roles of the insulin signaling pathway in temperature-mediated fat storage. This pathway is not only highly conserved from insects to mammals but also crucial in regulating lipid metabolism, cell proliferation, and tissue growth. The Forkhead box O (FoxO) protein functions as a key downstream signaling molecule in this pathway, mediating the inhibitory effects of insulin signaling. At elevated temperatures, direct targets of FoxO, such as *insulin receptor (InR)*, *Thor (Drosophila eukaryotic initiation factor 4E binding protein)*, and *FoxO* itself, are significantly upregulated, which indicates an inhibition of insulin signaling. Interestingly, this inhibition seems to occur independently of *Drosophila* insulin-like peptide (Ilp) stimuli, as not all Ilp transcripts were reduced at elevated temperatures. Furthermore, when S2R + *Drosophila* cells are incubated at high temperatures, there is a marked decrease in Akt phosphorylation, directly supporting the notion that elevated temperatures can inhibit insulin signaling in a cell-autonomous manner, independent of Ilp levels. Subsequent experiments demonstrated that either constitutively active InR or knockdown of FoxO prevents the reduction of body fat at high temperatures. Together, these findings highlight the critical role of the insulin signaling-FoxO branch in regulating lipid homeostasis under heat stress conditions.

## Introduction

Animals must allocate and adjust their energy and resources to effectively cope with the daily and annual cycles of both favorable and adverse conditions [1–3]. Temperature, a critical environmental factor, significantly influences the development, growth, and metabolism of all organisms [4–8]. For instance, an increase in temperature by 5–10°C significantly accelerates the development and regenerative ability of flatworms [8], yet lead to decreased egg production in fruit flies [9,10]. Furthermore, hyperthermia results in a reduction of lipid and carbohydrate storage in fruit flies [5,11]. Given that body weight changes are often resistant to environmental challenges [12], we are particularly interested in this pronounced reduction in body fat reserves and its underlying mechanism.

Thermal reaction norms for energy storage in fruit flies exhibit a second-degree polynomial function, displaying a reduction in both the size and total volume of adipose lipid droplets at both cold and warm temperatures [13]. This reduction is partially attributed to cellular damage and apoptosis in the fat body [11]. Importantly, the depletion of energy storage induced by thermal stress does not seem to be a passive process resulting from decreased food intake or increased metabolic rate. In fact, both food intake and metabolic rate rise proportionally with temperature [5], suggesting that active signaling mechanisms regulate the depletion of energy storage in response to thermal stress.

The insulin signaling pathway, crucial for regulating lipid and carbohydrate metabolism, is highly conserved across species from insects to mammals [14,15]. It has been suggested that temperature influences this pathway [16]. Insulin receptors (InRs), which consist of two extracellular α subunits and two transmembrane β subunits, initiate the insulin signaling cascade. Upon ligand binding, these tyrosine kinases undergo autophosphorylation, triggering a cascade of intracellular phosphorylation events [14]. This results in the phosphorylation of multiple tyrosine residues on the insulin receptor substrate (IRS) proteins, which then serve as binding sites and recruit various signaling molecules, including phosphatidyl-inositol-3 kinase (PI3K). Activated PI3K subsequently influences downstream kinases of the pathway, such as the Akt and mammalian target of rapamycin (mTOR) [14,17]. Akt activation results in the phosphorylation of forkhead box O (FoxO) transcription factor, which, when phosphorylated, is sequestered in the cytoplasm and is prevented from binding to target promoters, thereby reducing gene expression of its targets [18]. The critical role of insulin signaling is underscored by the fact that mutations in the InRs can lead to embryonic or early larval death in fruit flies, while viable hetero-allelic mutations often produce significantly smaller progeny [19].

Here, we demonstrate that high temperatures induce the depletion of body fat storage by inhibiting insulin signaling. Notably, this inhibition appears to be independent of *Drosophila* insulin-like peptide (Ilp) expression, as evidenced by the suppression of insulin signaling in S2R + *Drosophila* cells exposed to high temperatures. Furthermore, activating insulin signaling, either through the constitutively active form of InR or by the knockdown of FoxO, prevents the depletion of energy storage induced by thermal stress.

## Materials and methods

### Fly stocks

All fly stocks were maintained as previously described [20]. Briefly, flies were fed with Nutri-Fly BF food (Genesee Scientific) containing 1.59% yeast, 0.92% soy flower, 6.71% cornmeal, 6.71% corn syrup, 0.53% agar supplemented with 0.48% propionic acid, under 12-hour day and 12-hour night cycles at 24°C. UAS-constitutively active form of insulin receptor [21] (IR^CA^, Bloomington stock #8248), UAS-FoxO_RANi_ (Bloomington stock #27656), and *w*^*1118*^ (Bloomington stock #5905), lines were purchased from Bloomington stock center. The Act5c-Gal4 driver was a gift from Dr. John P. Masly (University of Oklahoma). The UAS/Gal4 binary transgene expression system was used to overexpress the constitutively active form of the insulin receptor (IR^CA^) and FoxO RNA_i_ (FoxO^KD^). The Gal4 transactivator was expressed under the control of the Act5C promoter (Act5C^P^), while IR^CA^ and FoxO^KD^ were under the control of the upstream activating sequence (UAS) (UAS-IR^CA^ and UAS-FoxO^KD^). Female Act5C-Gal4 flies were mated with male UAS-IR^CA^, UAS-FoxO^KD^, or *w*^*1118*^ flies to generate *Act5c > IR*^*CA*^, *Act5c > FoxO*^*KD*^, and *Act5c > w*^*1118*^. Two additional controls, *w*^*1118*^* > IR*^*CA*^ and *w*^*1118*^* > FoxO*^*KD*^, were created by mating female *w*^*1118*^ flies with male UAS-IR^CA^ and UAS-FoxO^KD^, respectively. The genotypes of the fly lines are listed in S1 Table.

### Triglyceride Analysis

Newly eclosed adult flies with the selected genotypes were collected and incubated for one to two weeks to allow for maturation. The matured flies were then subjected to experimental treatment before triglyceride (TG) analysis, following slight modifications to the method described [20]. Briefly, three to four sets of six adult flies of both sexes were collected, separated by sex, and homogenized with 180 μl of lysis buffer (PBS supplemented with 0.05% SDS). The lysates were heat-inactivated for 10 minutes at 65°C and centrifuged at 18,000g for 3 minutes to remove tissue debris. 4 μl of the resulting supernatant was transferred in a 96-well plate, mixed with Infinity solution (Thermo Fisher Scientific), incubated for 30 minutes at 37°C, and used to measure optical density at 500 nm. Final TG levels were normalized to the number of flies. All reagents used are listed in S2 Table.

### Cell culture

The *Drosophila melanogaster* S2R+ cells were cultured in Schneider’s *Drosophila* medium supplemented with 10% fetal bovine serum (FBS) and 1x penicillin-streptomycin-glutamine at 24°C as previously described [22]. Five million cells were seeded into each well of 6-well plates one day prior to the experiments. The next day, S2R+ cells were preincubated in nutrient-free media (NF-M) for one hour at 24°C or 31°C. Half of the wells remained in NF-M, while the other half were incubated with NF-M supplemented with 10 μg/ml insulin (Sigma-Aldrich, I0516) for an additional hour at their respective incubation temperatures. Phosphate buffered saline (PBS) was used as the NF-M.

### Western blotting and antibodies

Western blotting was performed according to standard procedures as described [22]. In brief, protein extracts of adult flies and S2R+ cells were prepared using PhosphoSafe Extraction Reagent (Sigma-Aldrich), supplemented with a protease inhibitor cocktail (Sigma-Aldrich), containing AEBSF, Aprotinin, Bestatin, E-64, Leupeptin, and Pepstatin A to inhibit phosphatases and proteases. Ten adult male flies were homogenized in 100μl of the extraction buffer, while S2R+ cells were homogenized in 25 μl of the same buffer. The homogenates were centrifuged, and the supernatant was collected, mixed with Laemmli sample buffer (Bio-Rad), and boiled. Equal amounts of protein were separated on an 8% SDS-polyacrylamide gel and transferred onto a 0.45μm nitrocellulose membrane. The membrane was blocked with LICORbio blocking buffer and then incubated with the indicated primary antibody. Subsequently, the membrane was washed with Tris-buffered saline (TBS) containing 0.1% tween 20 (TBST) and incubated with an HRP-conjugated secondary antibody. The Odyssey Fc imaging system (LICORbio) was used for chemiluminescent Western blot detection. All antibodies were diluted in LICORbio blocking buffer as follows: anti-phospho-Akt (Cell Signaling Technology, 4060, 1:1,000), anti-total Akt (Cell Signaling Technology, 4691,1:1,000), HRP-anti-rabbit (LICORbio, 926-80011,1:5,000). All Western blot experiments were repeated two to three times, with representative data shown.

### RNA extractions, reverse transcription, and qPCR

qPCR was performed following standard procedures as described [20]. For total RNA extraction, male *w*^*1118*^ flies were incubated at 24°C and 31°C for one week. Four flies were collected and immediately frozen at –20°C. The whole flies were then homogenized in Trizol (Thermo Fisher Scientific) using a pestle, following the manufacturer’s instructions. To generate cDNA, 1μg of total RNA was reverse-transcribed with Moloney Murine Leukemia Virus Reverse Transcriptase (Thermo Fisher Scientific). Gene expression was estimated by measuring the levels of transcripts, which were analyzed using Quantstudio 6 Flex (Applied Biosystems) with SYBR green master mix reagent (Apex Bioresearch) and specific primers as described previously [23,24] (Table 1). However, these primers may not be fully optimized to detect their targets or all gene isoforms. Gene expression values were normalized to the expression of ribosomal protein 49 (Rp49), an endogenous control, and calculated using the relative quantification method, determining C_T_ and ΔΔC_T_ values.

**Table 1:** Primer sequences for qPCR.

Primers	Sequences	Primers	Sequences
*Rp49-F*	CGATGTTGGGCATCAGATACT	*Rp49-R*	TGCTAAGCTGTCGCACAAAT
*ACC-F*	ATGAGCGAAACAAATGAGTCCA	*ACC-R*	GGAACTCGTCACATGCCTCG
*FASN-F*	TGACCAACAGTTCTTCGGTGT	*FASN-R*	GCGTCAATAATAGCTTCATGGGT
*Desat1-F*	CGAGTGATTCTGGTCATCTTCAA	*Desat1-R*	GGCGTCAGTCTCCGAGTAT
*FACL-F*	TGACCAACAGTTCTTCGGTGT	*FACL-R*	GCGTCAATAATAGCTTCATGGGT
*Brum-F*	GTCTCCTCTGCGATTTGCCAT	*Brum-R*	CTGAAGGGACCCAGGGAGTA
*HSL-F*	GAGTACGATTTTGACGAGCAGA	*HSL-R*	GCAGATGCTAATGCCATGCG
*SREBP-F*	ACCAACAGCCACCATACATCA	*SREBP-R*	AGACAAAGCTACTGCCCAGAG
*InR-F*	GAAGTGGAGACGACGGGTAAA	*InR-R*	TCGCGCTGTTGTCGATTGTT
*Thor-F*	CAGATGCCCGAGGTGTACTC	*Thor-R*	CATGAAAGCCCGCTCGTAGA
*FoxO-F*	CATGGGGAAATCTATCCTATGCG	*FoxO-R*	ACTCAGTGTCAATCGTTTGTCG
*Ilp1-F*	AATGGCAATGGTCACGCCGACTGG	*Ilp1-R*	GCTGTTGCCCAGCAAGCTTTCACG
*Ilp2-F*	AGCAAGCCTTTGTCCTTCATCTC	*Ilp2-R*	ACACCATACTCAGCACCTCGTTG
*Ilp3-F*	TGTGTGTATGGCTTCAACGCAATG	*Ilp3-R*	CACTCAACAGTCTTTCCAGCAGGG
*Ilp4-F*	TGGATTTACACGCCGTGTCAGGCG	*Ilp4-R*	ACACCCTTCTCCGTATCCGCATGG
*Ilp5-F*	GAGGCACCTTGGGCCTATTC	*Ilp5-R*	CATGTGGTGAGATTCGGAGC
*Ilp6-F*	TGCTAGTCCTGGCCACCTTGTTCG	*Ilp6-R*	GGAAATACATCGCCAAGGGCCACC
*Ilp7-F*	GAGCTGTACTCCTGTTCGTCCTGC	*Ilp7-R*	TCCAAGCCTCATCATTGCCCGTCC
*Ilp8-F*	CGACAGAAGGTCCATCGAGT	*Ilp8-R*	GATGCTTGTTGTGCGTTTTG

## Results

### High ambient temperatures reduce body fat in *Drosophila melanogaster*

**Fig 1:**
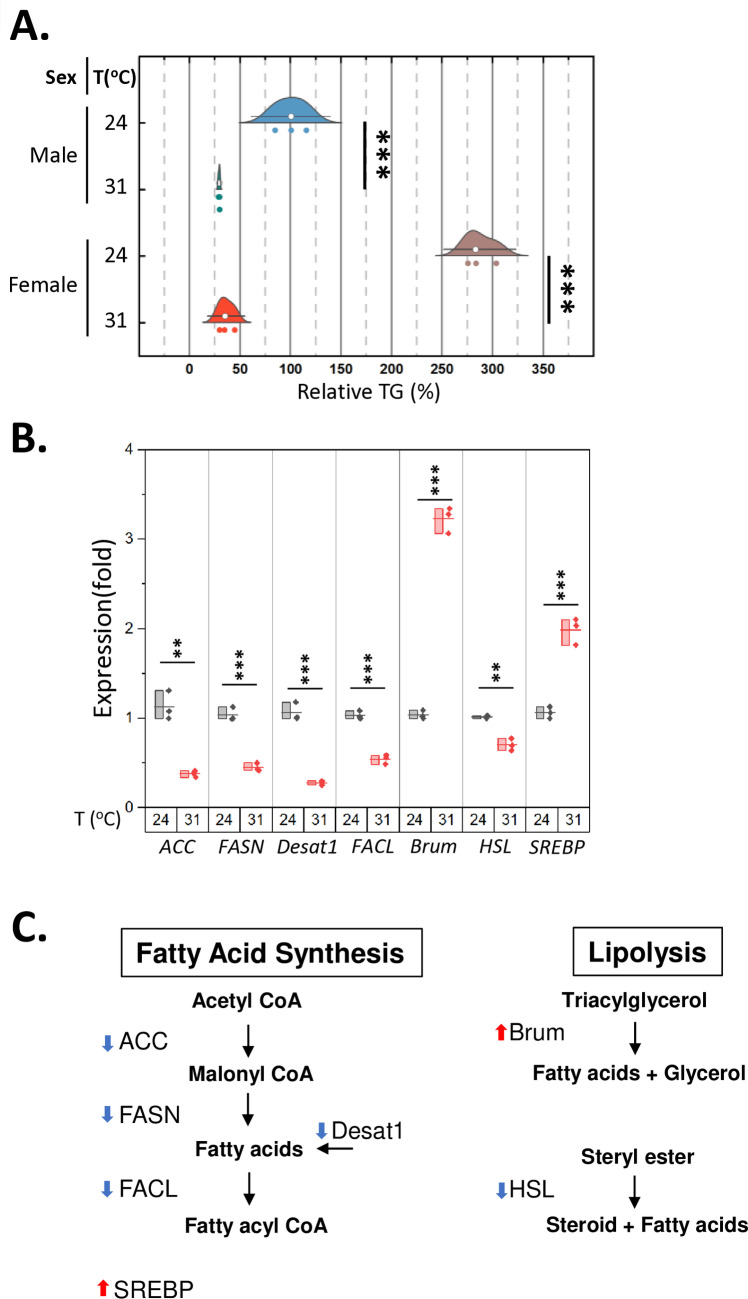
High ambient temperatures reduce body fat and alter the expression of genes involved in lipid metabolism. **A** *w*^*1118*^ flies aged 1–2 weeks old were incubated at two different incubation temperatures, 24°C and 31°C, for seven days. Three sets of six flies were sorted into microcentrifuge tubes for the triglyceride assay. The male body fat content at 24°C was set to 100, and the rest of the measurements were calculated relative to the male body fat. Relative Body Fat (%) =  (TG_:Experimental group_/TG_*: w*_^*1118*^_*male at 24*°C_) X 100. In the raincloud plot, open circles represent the median, with whiskers indicating the 95% confidence interval and individual data points are shown as dots. Statistical differences among the four groups (Male-24°C, Male-31°C, Female-24°C, and Female-31°C) were evaluated using a one-way ANOVA with Tukey’s HSD post hoc tests. *** p < 0.001. Three replicates per group, six flies in each replicate. **B.** The expression of genes involved in lipid metabolism was determined by qPCR. In the box plots, the boxes represent the data range; the horizontal lines within the boxes indicate the average; individual data points are shown as dots. Statistical analysis was conducted using t-test. ** p < 0.01; *** p < 0.001. Four male *w*^*1118*^ flies, aged 1–2 weeks, per each group. **C.** The diagram illustrates the metabolic pathways for fatty acid synthesis and lipolysis, with arrows indicating expression changes at 31°C. Acetyl-CoA carboxylase (ACC, CG11198) generates malonyl Co-A (the rate-limiting substrate of fatty acid synthesis); fatty acid synthase (FASN, CG3523) generates fatty acids by condensing acetyl Co-A and malonyl Co-A; stearoyl-CoA desaturase (Desat1, CG5887) catalyzes the formation of monounsaturated fatty acids; fatty acyl Co-A ligase (FACL, CG6178) catalyzes thioesterification between fatty acids and CoA for fatty acid metabolism; brummer (brum, CG5295) is the lipid droplet associated triacylglycerol lipase; hormone-sensitive lipase (HSL, CG11055) regulates steryl ester hydrolysis; sterol regulatory element binding protein (SREBP, CG8522) is a major transcriptional regulator of lipid metabolism. All source data for the graphs are presented in the supplementary data 2.

Ambient temperature governs a wide range of physiological processes in animals, including development, reproduction, and energy metabolism [4–8]. To better understand the effects of high ambient temperature on energy homeostasis, we subjected *w*^*1118*^ flies to two different conditions: a higher temperature of 31°C and an optimum temperature of 24°C for seven days. Subsequently, we measured their overall body triglyceride levels using a colorimetric method [20]. High ambient temperature substantially reduced fat storage in both males and females, with females showing a higher rate of overall fat reduction (Fig 1A, S3 Table). Consequently, the levels of body fat between males and females become comparable at 31°C (Fig 1A), abolishing the sexual dimorphism of body fat [25]. This is supported by a significant temperature-sex interaction, as indicated by a two-way ANOVA (p <  0.0001) (S4 Table). To gain further insights into the mechanisms underlying the reduction in fat storage at high temperatures, we measured the expression levels of genes responsible for regulating triglyceride synthesis and mobilization[24]. The expression of genes related to fatty acid synthesis exhibited a significant decrease, including *Acetyl-CoA carboxylase (ACC), fatty acid synthase (FASN), stearoyl-CoA desaturase (Desat1), and long-chain fatty acid-CoA ligase (FACL)*. However*, brummer (Brum)*, a key triglyceride lipase, was significantly upregulated, while *hormone-sensitive lipase (HSL)*, a steryl ester hydrolysis enzyme [26], was downregulated (Fig 1B). These results support the notion that high ambient temperatures reduce body fat by inhibiting fatty acid synthesis and increasing triglyceride hydrolysis (Fig 1C). Notably, the *sterol regulatory element binding protein (SREBP)*, an essential transcription factor for lipid homeostasis, was significantly upregulated, likely as a compensatory response to maintain lipid metabolism balance following substantial changes in lipid metabolism gene expression.

### High ambient temperatures suppress the insulin signaling pathway

**Fig 2:**
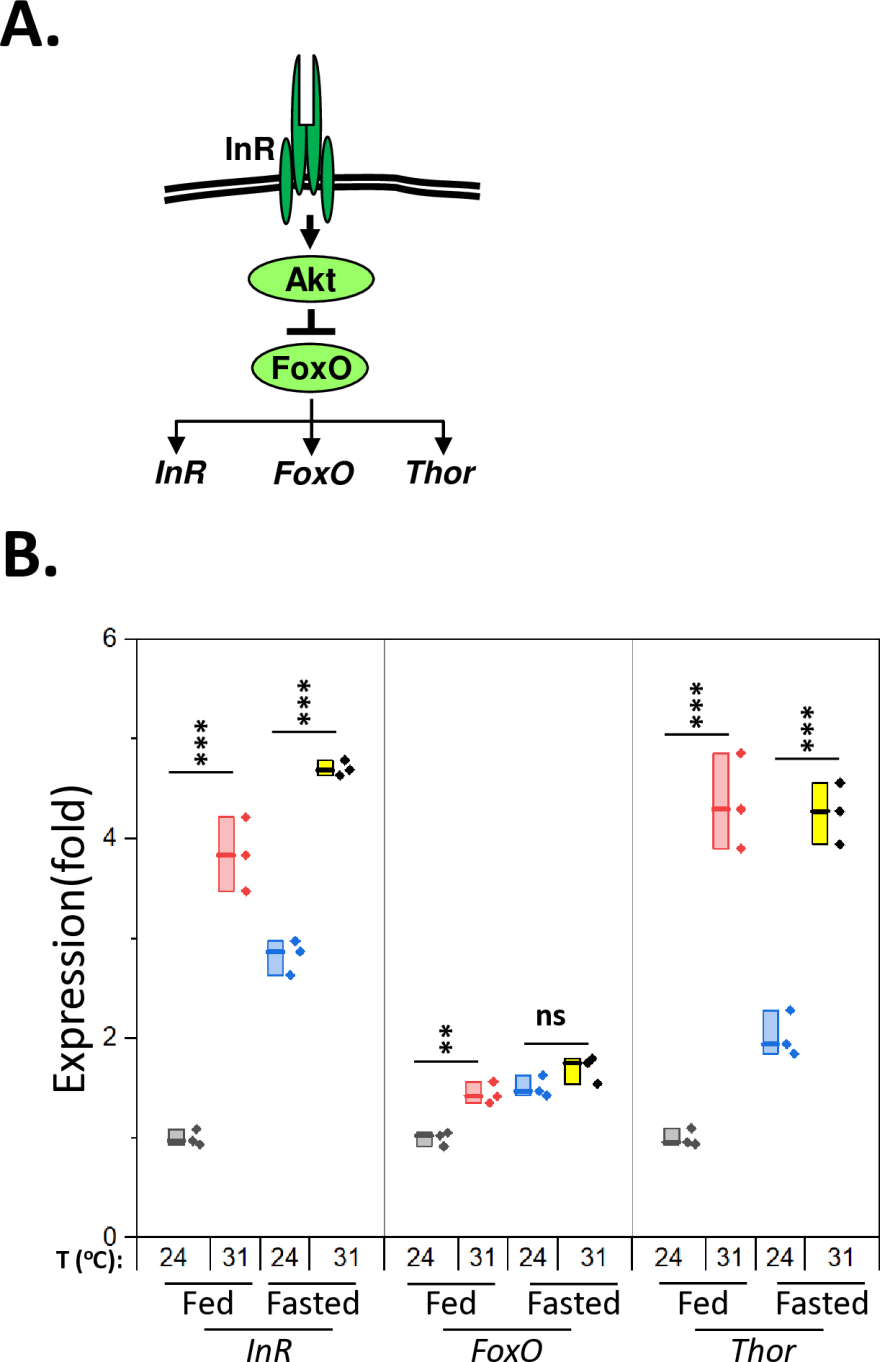
High temperatures regulate the insulin signaling pathway. **A.** Diagram of the insulin signaling pathway and FoxO’s target genes. **B.** *w*^*1118*^ flies were incubated at two different temperatures, 24°C or 31°C, for seven days. Then, half of the flies were fasted for one day. The expression levels of *lnR, FoxO*, and *Thor* were determined using qPCR among the four groups (Fed-24°C, Fed-31°C, Fasted-24°C, and Fasted-31°C). Statistical differences were analyzed using a one-way ANOVA with Tukey’s HSD post hoc tests. In the box plots, the boxes represent the data range; the horizontal lines within the boxes indicate the median; individual data points are shown as dots. ** p < 0.01; *** p < 0.001. Four male *w*^*1118*^ flies, aged 1–2 weeks, per each group. *Insulin receptor (InR), Akt/protein kinase* ***B*** *(PKB), Forkhead box* ***O*** *(FoxO),* and *Eukaryotic initiation factor 4E binding protein (Thor).*

Given the critical role of the insulin signaling pathway in lipid and carbohydrate metabolism [14], we hypothesized that the flies reared at high ambient temperatures would show reduced insulin signaling, leading to a substantial reduction in triglyceride storage. When the insulin signaling pathway is activated, the FoxO transcription factor is phosphorylated and sequestered in the cytoplasm, resulting in the inhibition of its target gene expression [14]. We thus assessed insulin signaling by measuring the expression of FoxO target genes, including *insulin receptor (lnR), FoxO* itself, and *Thor (Drosophila eukaryotic initiation factor 4E binding protein)* (Fig 2A). When *w*^*1118*^ flies were fasted to suppress insulin signaling, the expression of FoxO’s target genes was significantly upregulated. Similarly, elevated ambient temperature (31°C) significantly increased the expression of the FoxO target genes regardless of nutritional state, except for *FoxO* expression under fasting condition, indicating that high temperatures may suppress the insulin signaling pathway (Fig 2B, S5–S7 Tables). To investigate the interaction between temperature and nutritional state, we analyzed *InR* and *Thor* expression using a two-way ANCOVA with the *FoxO* as a covariate. There was a significant interaction between temperature and nutritional state for *InR* (p = 0.04), but not for *Thor* (p = 0.07) levels (S8 Table).

### The expression of all *Ilps* is not reduced by high ambient temperatures

**Fig 3:**
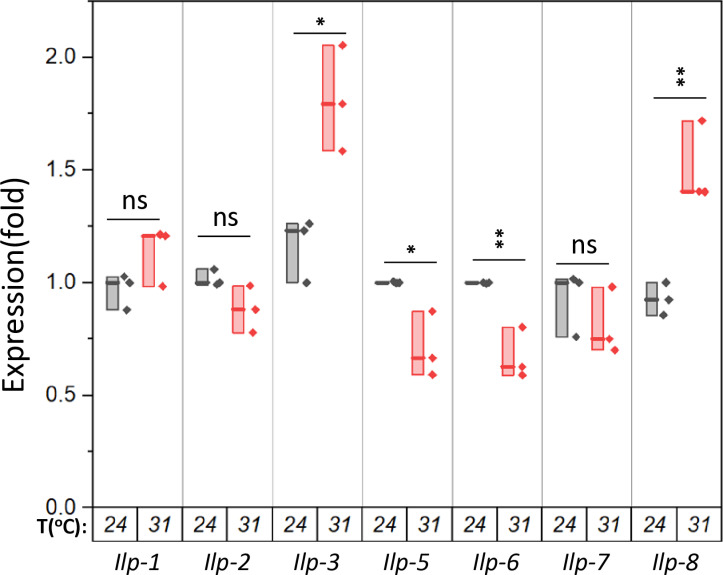
High temperatures do not reduce the expression of *all insulin-like peptides* (*Ilps*). Male *w*^*1118*^ flies were incubated for one week at either 24°C or 31°C, for seven days. cDNA synthesized from whole-body total RNA was used to determine the levels of *Ilps*. In the box plots, the boxes represent the data range; the horizontal lines within the boxes indicate the median; individual data points are shown as dots. Four male flies, aged 1–2 weeks, per each group. Statistical analysis was performed by t-test. *p < 0.05; ** p < 0.01.

The *Drosophila* genome encodes eight insulin-like peptides (Ilps) [27]. To better understand how high temperatures inhibit insulin signaling, we measured the expression of Ilps in adult flies using qPCR and detected transcripts for seven *Ilps*. We observed significant reductions in the expression of *Ilp-5* and *Ilp-6*, while *Ilp-3* and *llp-8* showed increased expression. However, the expression of the other three *Ilps* (*Ilp-1, Ilp-2*, and *Ilp-7*) remained largely unchanged (Fig 3). *Ilp-1, Ilp-2, Ilp-3,* and *Ilp-5* are primarily produced from the insulin-producing cells in the brain and are involved in regulating growth and carbohydrate metabolism [28]. Notably, *Ilp-2*, the closest homolog to human insulin, is induced by cold temperatures and has been shown to promote larval development and growth [15,23]; however, it was only marginally reduced by high temperatures (Fig 3). Although protein levels of Ilps in the hemolymph still need to be determined, the decreased expression of *Ilps* alone may not fully account for the observed reduction in insulin signaling in the organism.

### The fasting-refeeding regimen at high temperatures activated the insulin signaling pathway

**Fig 4:**
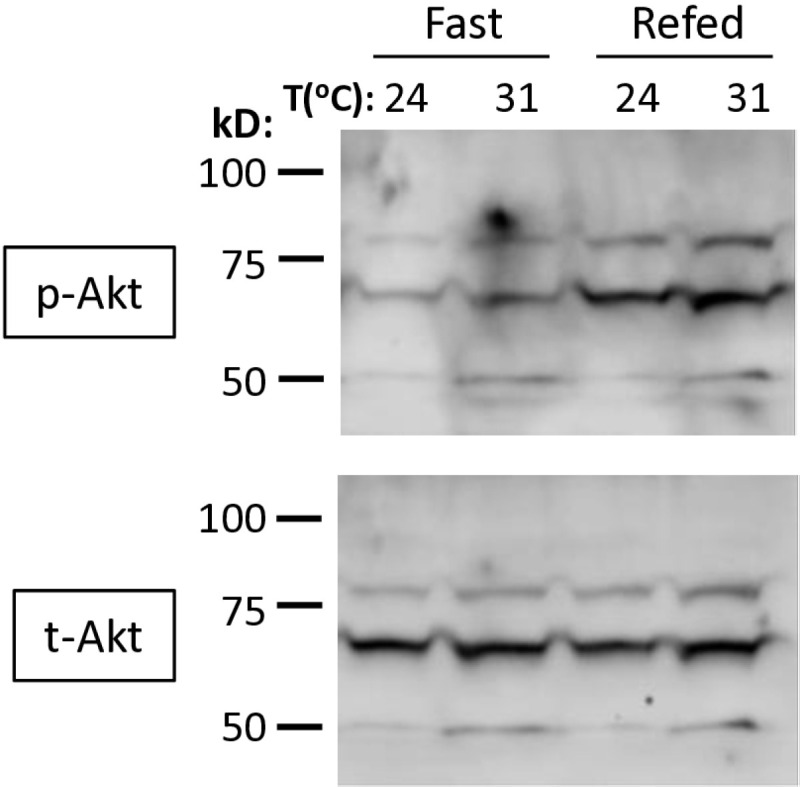
The fasting-refeeding regimen increased Akt phosphorylation. Male *w*^*1118*^ flies were incubated at two different temperatures, 24°C or 31°C, for three days followed by one day of fasting. Half of the flies were kept fasting, while the other half were refed for 2 hours at their initial incubation temperatures. Then, flies were collected from each condition, and protein extracts were prepared for analysis. Ten flies, aged 1–2 weeks, per each group. Phosphorylated Akt (p-Akt) and total Akt (t-Akt) were detected by Western blot. Statistical analysis was not performed due to the small sample sizes.

We further tested whether high ambient temperatures inactivate the insulin signaling pathway and render it unresponsive to any stimuli. Typically, insulin signaling is activated by refeeding following a period of fasting [29]. To test this response, *w*^*1118*^ flies were fasted at 31°C for one day and then refed before preparing their protein extracts. Akt phosphorylation was assessed by Western blots as a measure of pathway responsiveness. Upon refeeding, the level of Akt phosphorylation at 31°C was increased to a level comparable to that at 24°C (Fig 4), indicating that the insulin signaling pathway remains functional under high-temperature conditions. However, this Western blot data should be interpreted with caution due to small sample sizes and methodological constrains.

### Cells can autonomously suppress insulin signaling at high temperatures

**Fig 5:**
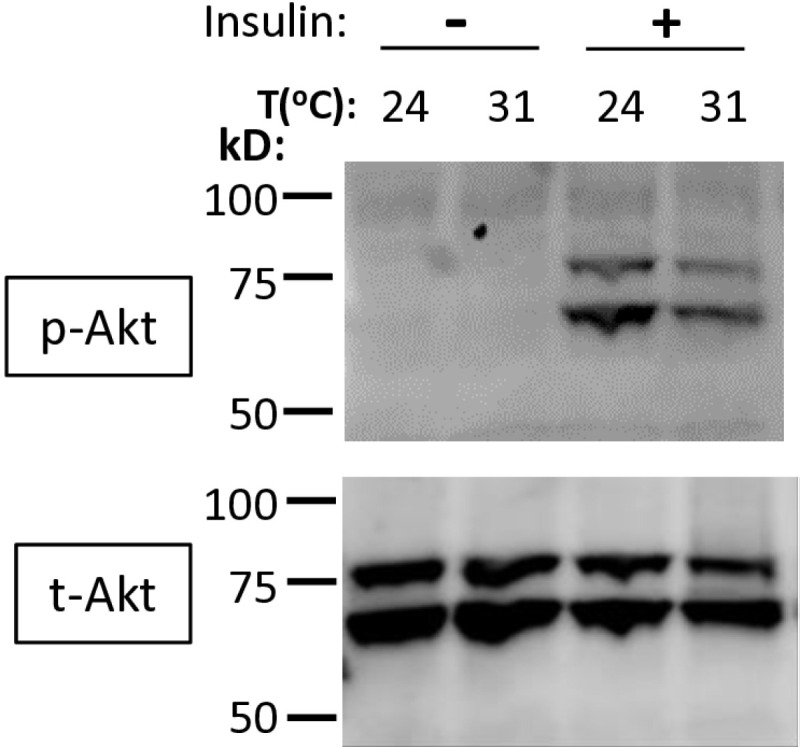
Elevated temperatures inhibit insulin signaling in S2R+ *Drosophila* cells. The same number of S2R+ cells in 6-well plates were preincubated in nutrient-free media (NF-M) at 24°C or 31°C for 1 hour. Half of the wells remained in NF-M, while the other half were incubated with NF-M supplemented with 10 μg/ml insulin for an additional hour at their respective preincubated temperatures. Total protein extracts from S2R+ cells were prepared and analyzed by Western blot using antibodies for phosphorylated Akt (p-Akt) and total Akt (t-Akt).

To further investigate whether high temperatures affect the insulin signaling pathway independently of Ilp ligand levels, we incubated S2R+ *Drosophila* cells at two different temperatures. We assessed the pathway’s activity using Western blot, and basal levels of Akt phosphorylation were achieved in nutrient-free media (NF-M). When the cells were incubated in NF-M supplemented with insulin, Akt phosphorylation was inhibited at 31°C compared to at 24°C, indicating suppression of insulin signaling at higher temperatures (Fig 5). Quantification of phosphorylated Akt relative to total Akt showed only a trend toward reduction but did not reach statistical significance (S1 Fig). Notably, this approach is subject to inherent methodological constraints in accurately estimating variances across multiple blots, largely due to the limited number of samples per blot.

### The insulin signaling pathway is responsible for high temperature-mediated body fat loss via FoxO

**Fig 6:**
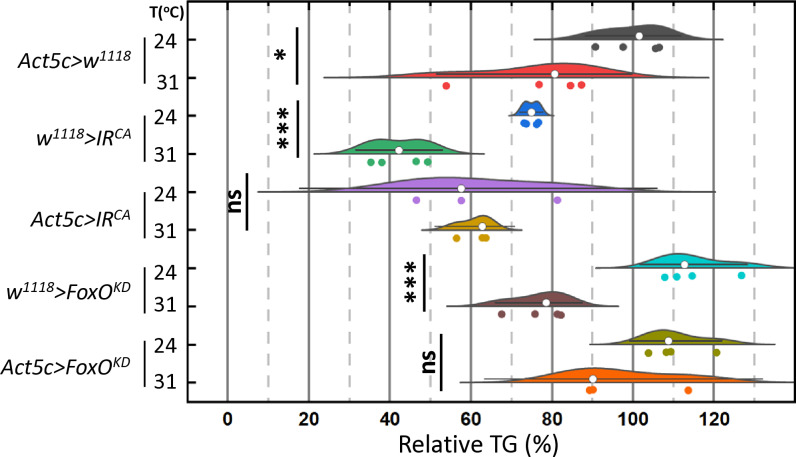
The constitutively active form of insulin receptor and knockdown of FoxO block high temperature-mediated body fat loss. One-week-old female flies expressing a constitutively active form of the insulin receptor (*Act5c > IR*^*CA*^) and *FoxO* RNA_i_ (*Act5c > FoxO*^*KD*^), along with control flies, were incubated at 24°C and 31°C for seven days. Triglyceride (TG) levels were then determined using a colorimetric method and normalized to the number of flies. The TG content of each experimental group was compared to that of control flies (*Act5c > w*^*1118*^), which was set to 100%. The relative amount of body fat (%) was calculated using the following formula. Relative Body Fat (%) =  (TG_: Experimental group_/TG_: Act5c > *w*_^*1118*^_, female *at 24*_°_C_) X 100; In the raincloud plot, open circles represent the median, with whiskers indicating the 95% confidence interval, and individual data points are shown as dots. Statistical differences among the two temperature groups in each genotype were evaluated using t-test. *  p < 0.05, ** p < 0.01, *** p < 0.001. Three or four replicates per group, six flies in each replicate.

To investigate the roles of the insulin signaling pathway on high temperature-mediated body fat loss, we conducted experiments to test if activating this pathway could abrogate the effect. We generated a constitutively active form of insulin receptor flies (*Act5c > IR*^*CA*^) by combining the ubiquitous Gal4 driver, *Act5c-Gal4*, and a constitutively active form of insulin receptor (IR^CA^) [21]. Similarly, we generated control flies, by mating Act5c-Gal4 with *w*^*1118*^ (*Act5c > w*^*1118*^) and *w*^*1118*^ with IR^CA^ (*w*^*1118*^* > IR*^*CA*^). The flies expressing the constitutively active form of the insulin receptor (*Act5c > IR*^*CA*^) and their two controls (*Act5c > w*^*1118*^ and *w*^*1118*^* > IR*^*CA*^) were incubated at 24°C and 31°C for seven days. Overexpression of the constitutively active form of the insulin receptor completely blocks high temperature-mediated body fat loss (Fig 6). We further tested whether this effect is mediated through the FoxO branch, a downstream transcription factor of the insulin signaling pathway. Knockdown of FoxO (*Act5c > FoxO*^*KD*^) prevents body fat loss triggered by high temperatures (Fig 6), highlighting the essential role of the insulin signaling-FoxO axis on high temperature-mediated body fat loss. As predicted, a two-way ANOVA reveals that the interaction between temperature and genotype significantly affects body fat content (S9 Table). Subsequent t-tests comparing the two temperature conditions within each genotype revealed that elevated temperature did not reduce body fat in either *Act5c > IR*^*CA*^ or *Act5c > FoxO*^*KD*^ flies (Fig 6).

## Discussion

Ambient temperatures influence every aspect of animal physiology, from growth and development to energy metabolism [5,7,16]. Ectothermic animals, such as invertebrates and fish, experience significant changes in body temperature with environmental fluctuations, while endothermic animals maintain a constant body temperature. Although ectothermic animals are affected by fluctuating temperatures more severely [30–32], both types of animals have developed behaviors and physiological adaptations to lessen the metabolic impact of these changes. These adaptations involve reducing activity and inducing dormancy in the cold, as well as increasing water intake and seeking shade during hot conditions. Additionally, animals can adjust the thermal sensitivity of their enzymes and signaling pathways, modifying the thermal performance curve to buffer against negative impacts on their metabolic processes [33,34].

Here, we demonstrated that high temperatures inhibit the insulin signaling pathway, leading to body fat loss in fruit flies. But how exactly does temperature suppress the insulin signaling pathway? As shown in Fig 3, not all Ilps, at least their mRNAs, are reduced by high temperatures, and the insulin signaling pathway remains responsive at high temperatures (Fig 4). Additionally, S2R+ *Drosophila* cells reduce Akt phosphorylation at high incubation temperatures (Fig 5), indicating that high temperatures alone are sufficient to inhibit insulin signaling. However, the redundant and complementary roles of Ilps in metabolism, as well as their dynamic temporal and spatial expression during development, suggest that llps may still play a role in the temperature-mediated suppression of insulin signaling *in vivo* [35–38]. Another potential mechanism for this inhibition is crosstalk between high temperatures and other nutrient-sensing pathways, such as mTOR [16,22,39,40], supported by the significant interaction between temperature and nutritional state on *InR* expression (S8 Table).

Given that the genetic background is an important factor in determining body fat content [11,41], it is not surprising that animals with mixed backgrounds, including *Act5c > w*^*1118*^*, w*^*1118*^*> IR*^*CA*^, and *w*^*1118*^*>  FoxO*^*KD*^, are less sensitive to high temperature-mediated body fat loss. Therefore, backcrossing each line to *w*^*1118*^ or another common background strain would help assess the effects of insulin signaling on temperature-mediated body fat loss without genetic background variability. Interestingly, male flies with a mixed background have not responded to high temperature-mediated body fat loss (S2 Fig) while female flies with these mixed genetic backgrounds were responsive to heat-mediated body fat loss. We speculate that this sexual difference may arise from the differential expression of insulin signaling pathway genes, which are shown to be higher in females [42,43].

The inhibition of the insulin signaling pathway under high temperatures would be advantageous to flies as it pauses development, growth, and energy reserve accumulation while channeling extra energy towards maintenance during thermal stress. Possibly, inhibition of insulin signaling could reduce reactive oxygen species (ROS), which can damage DNA and cellular organelles [44]. Uncoupling proteins (UCPs) are mitochondrial transmembrane proteins that regulate ROS and the proton gradient. UCP4 reduces ROS formation in the central nervous system, while UCP1 produces heat in brown adipose tissue by dissipating the proton gradient [45]. In fruit flies, the UCP4 ortholog, UCP4C, is upregulated when insulin signaling is inhibited [46] and subsequently reduces elevated ROS caused by high ambient temperatures [47]. Thus, inhibition of the insulin signaling pathway at high temperatures provides an advantage to flies, explaining why they adopt this strategy.

The transient receptor potential (TRP) family of cation channels is primarily expressed in neuronal tissues and detects temperature, controlling thermal preference behaviors in animals [48]. However, our data suggest that non-neuronal cells may have a thermosensation mechanism linking ambient temperature to insulin signaling (Fig 5). The endoplasmic reticulum (ER), a membranous network in the cytoplasm, is the site of protein modification, lipid synthesis, and Ca^2+^ storage [49]. Elevated temperatures, protein synthesis overload, or Ca^2+^ imbalance can cause stress in the ER and lead to cellular dysfunction, inflammation, and obesity [49–51]. We postulate that major ER transmembrane proteins can sense high temperatures and regulate insulin signaling once activated by heat shock proteins and unfolded proteins [49,52–54].

## Supporting information

S1 File Raw images. (PDF)

S2 File Source data for graphs. (XLSX)

S1 Table The genotypes of the fly lines used in this study. (TIF)

S2 Table The reagents used in this study. (TIF)

S3 Table Results of one-way ANOVA for body fat differences by temperature and sex combination. Statistical differences among the four temperature and sex combination groups were evaluated using a one-way ANOVA, followed by Tukey’s HSD post hoc tests (SPSS, version 26). The groups were M24 (Male-24°C), M31 (Male-31°C), F24 (Female-24°C), and F31 (Female-31°C).
(TIF)

S4 Table Results of two-way ANOVA for body fat differences by temperature and sex interaction. (TIF)

S5 Table Results of one-way ANOVA for *InR* expression differences by temperature and nutritional state combination. Statistical differences among the four temperature and nutritional state combination groups were evaluated using a one-way ANOVA, followed by Tukey’s HSD post hoc tests (SPSS, version 26). The groups were Fast24 (Fasted-24°C), Fast31 (Fasted-31°C), Fed24 (Fed-24°C), and Fed31 (Fed-31°C).
(TIF)

S6 Table Results of one-way ANOVA for *FoxO* expression differences by temperature and nutritional state combination. Statistical differences among the four temperature and nutritional stage combination groups were evaluated using a one-way ANOVA, followed by Tukey’s HSD post hoc tests (SPSS, version 26). The groups were Fast24 (Fasted-24°C), Fast31 (Fasted-31°C), Fed24 (Fed-24°C), and Fed31 (Fed-31°C).
(TIF)

S7 Table Results of one-way ANOVA for *Thor* expression differences by temperature and nutritional state combination. Statistical differences among the four temperature and nutritional stage combination groups were evaluated using a one-way ANOVA, followed by Tukey’s HSD post hoc tests (SPSS, version 26). The groups were Fast24 (Fasted-24°C), Fast31 (Fasted-31°C), Fed24 (Fed-24°C), and Fed31 (Fed-31°C).
(TIF)

S8 Table Results of two-way ANCOVA for *InR* and *Thor* expression differences by temperature and nutritional state interaction with *FoxO* as a covariate. (TIF)

S9 Table Results of two-way ANOVA for body fat differences by temperature and genotype interaction. (TIF)

S1 Fig Densities of pAkt/tAkt bands from Western blots were quantified. S2R+ cells were plated in 6-well plates and preincubated in nutrient-free media (NF-M) at 24°C or 31°C for 1 hour. Half of the wells remained in NF-M, while the other half were incubated with NF-M supplemented with 10 μg/ml insulin for an additional hour at their respective preincubated temperatures. Total protein extracts from S2R+ cells were prepared and analyzed by Western blot using antibodies specific to phosphorylated Akt (p-Akt) and total Akt (t-Akt). Images from three replicates (Fig 5 raw images) were analyzed using imageJ software to measure the densities of both p-Akt and t-Akt bands. Open squares represent the mean of pAkt/tAkt values, lines indicate the median, and individual data points are shown as dots.
(TIF)

S2 Fig Male flies with a mixed background have not responded to high temperature-mediated body fat loss. One-week-old male flies expressing a constitutively active form of the insulin receptor (*Act5c > IR*^*CA*^) and *FoxO* RNA_i_ (*Act5c > FoxO*^*KD*^), along with control flies, were incubated at 24°C and 31°C for seven days. Triglyceride (TG) levels were then determined using a colorimetric method and normalized to the number of flies. The TG content of each experimental group was compared to that of control flies (*Act5c > w*^*1118*^), which was set to 100%. The relative amount of body fat (%) was calculated using the following formula. Relative Body Fat (%) =  (TG_: Experimental group_/TG_: Act5c > *w*_^*1118*^_, female *at 24*°C_) X 100; Open squares represent the mean, lines indicate the median, and individual data points are shown as dots. Statistical differences among the two temperature groups in each genotype were evaluated using t-test. *  p < 0.05, ** p < 0.01. Three or four replicates per group, six flies in each replicate.
(TIF)
