# Supplementary figures and images for: Elevated ambient temperature reduces fat storage through the FoxO-mediated insulin signaling pathway
Source: PLoS One. 2025 Feb 26;20(2):e0317971. doi: 10.1371/journal.pone.0317971 (PMC11864546; doi:10.1371/journal.pone.0317971)

Fig 4 Raw Image

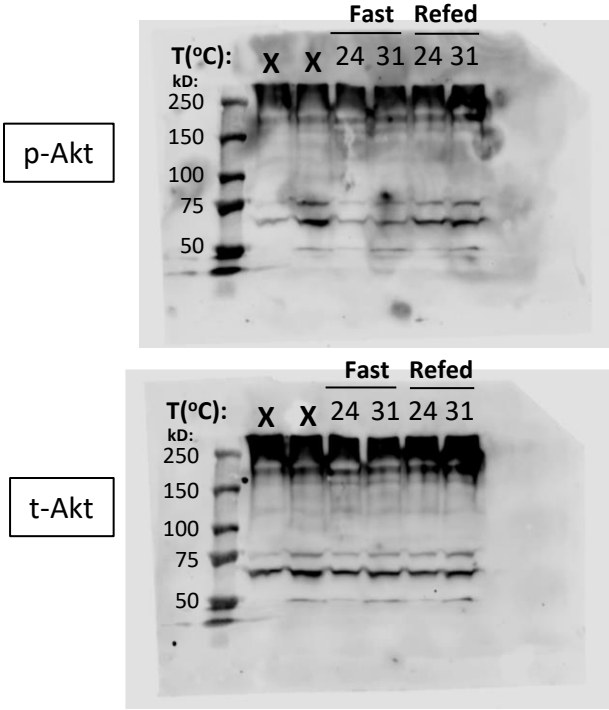

Male *w<sup>1118</sup>*

Fig 4 Image-2

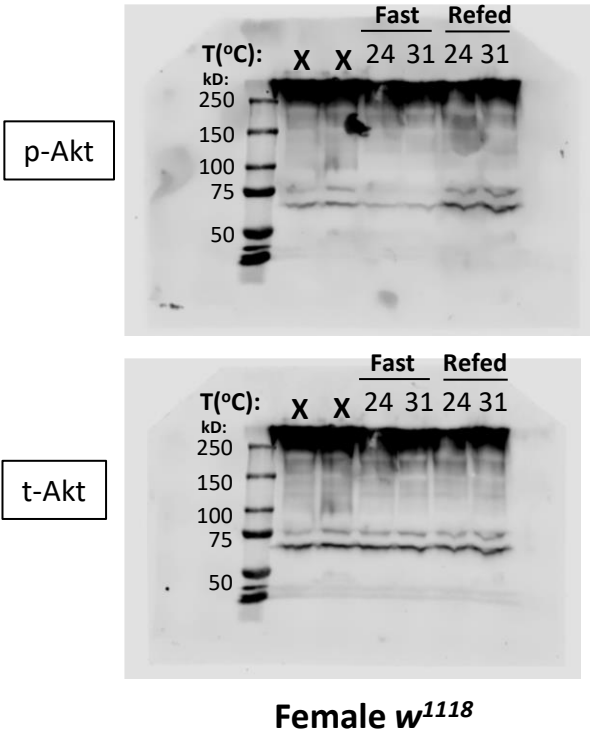

Fig 5 Raw Image

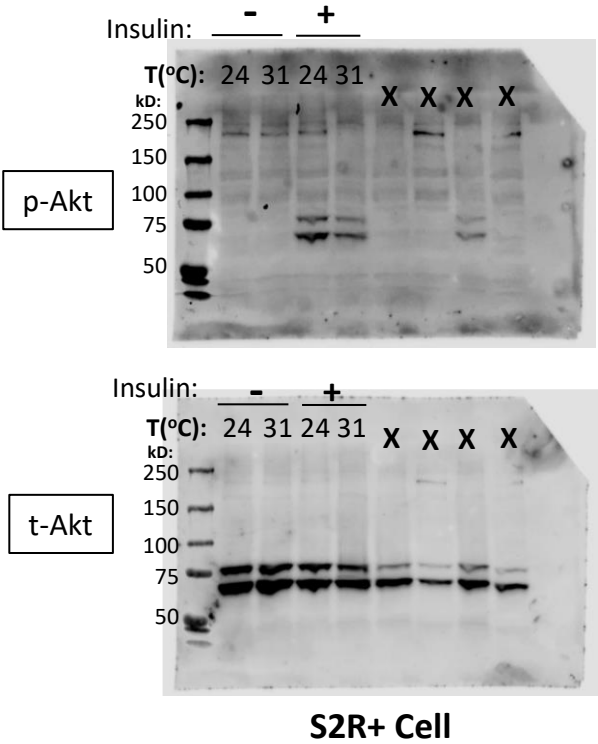

Fig 5 image 2

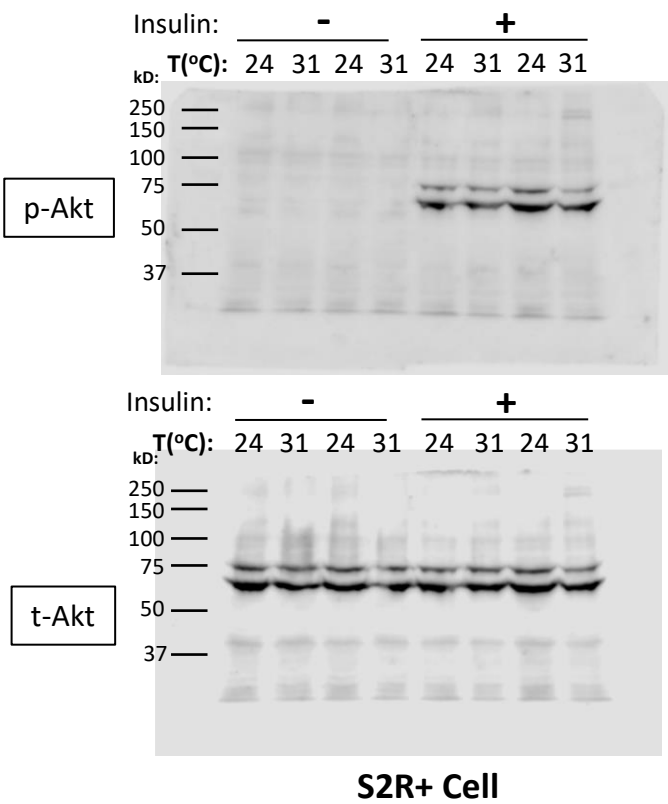

Supplement: S1 File — (PDF) [file pone.0317971.s001.pdf]

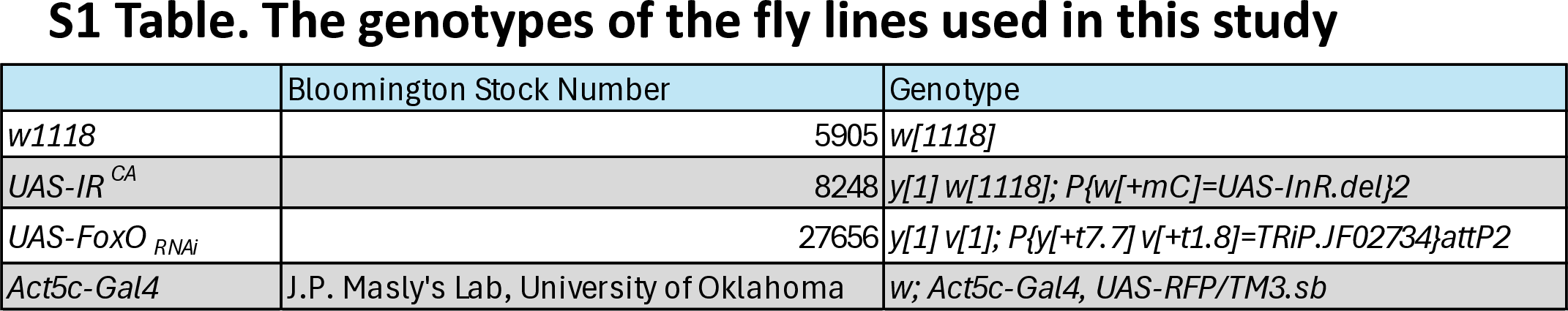

Supplement: S1 Table — (TIF) [file pone.0317971.s003.tif]

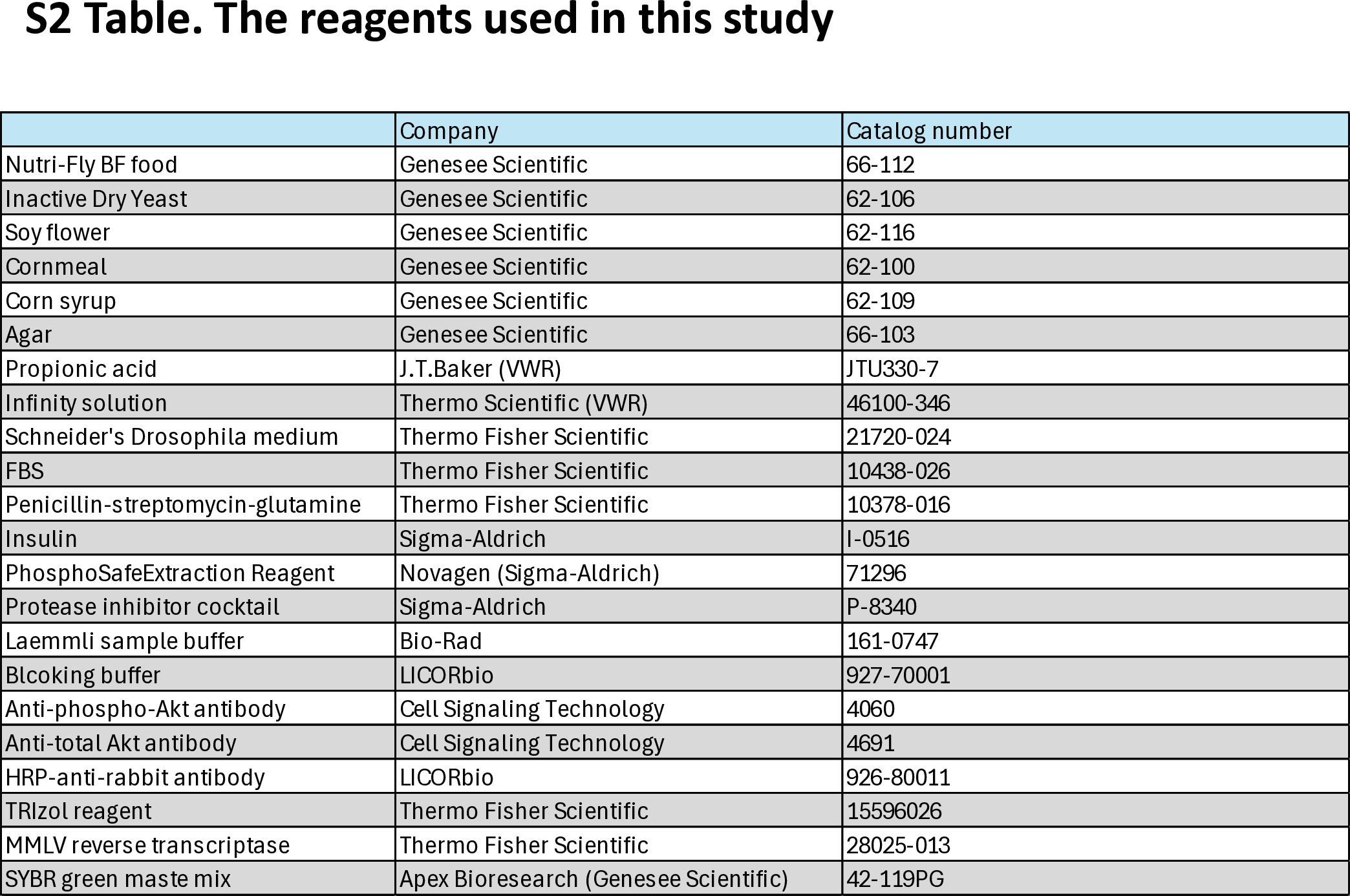

Supplement: S2 Table — (TIF) [file pone.0317971.s004.tif]

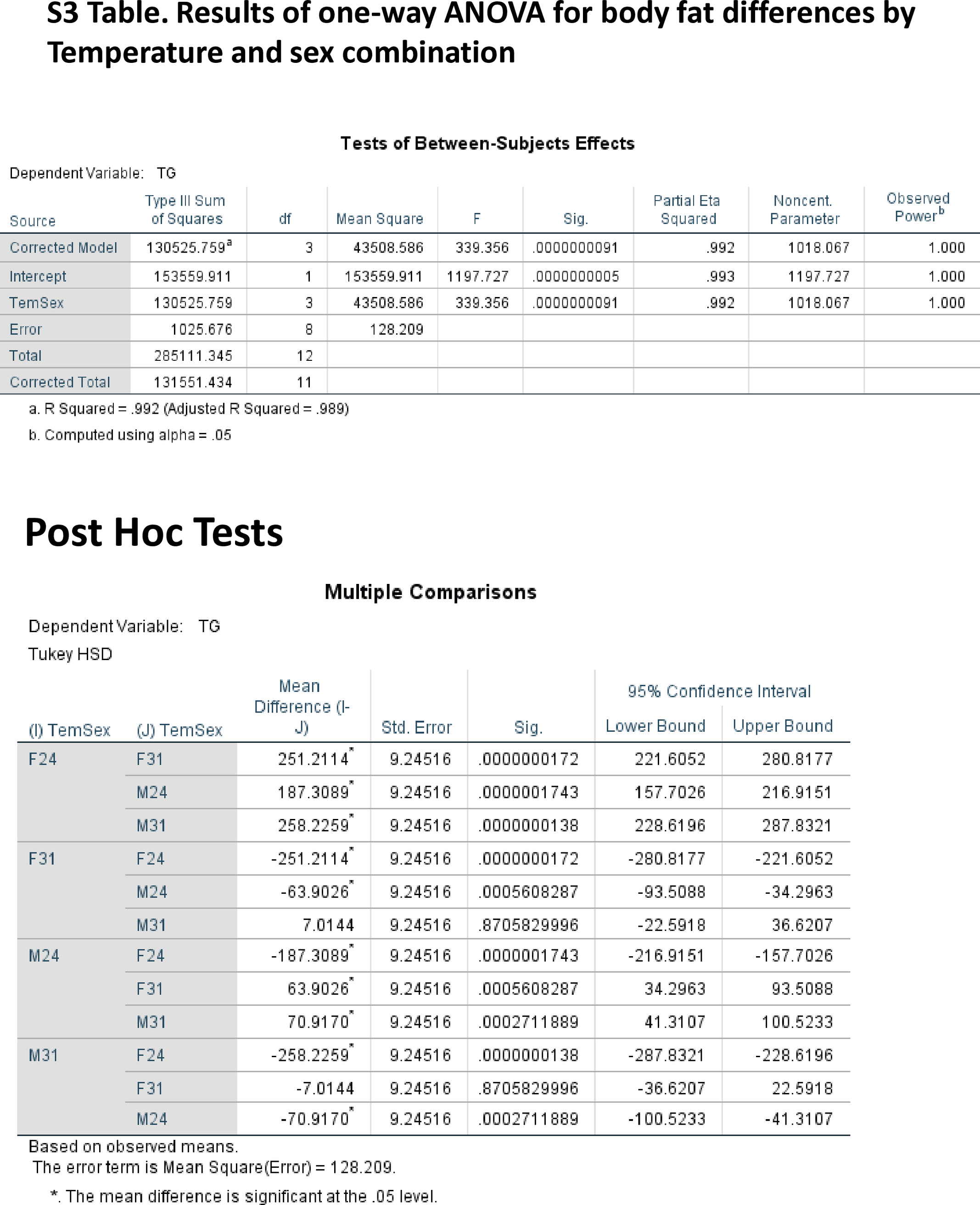

Supplement: S3 Table — Statistical differences among the four temperature and sex combination groups were evaluated using a one-way ANOVA, followed by Tukey’s HSD post hoc tests (SPSS, version 26). The groups were M24 (Male-24°C), M31 (Male-31°C), F24 (Female-24°C), and F31 (Female-31°C). (TIF) [file pone.0317971.s005.tif]

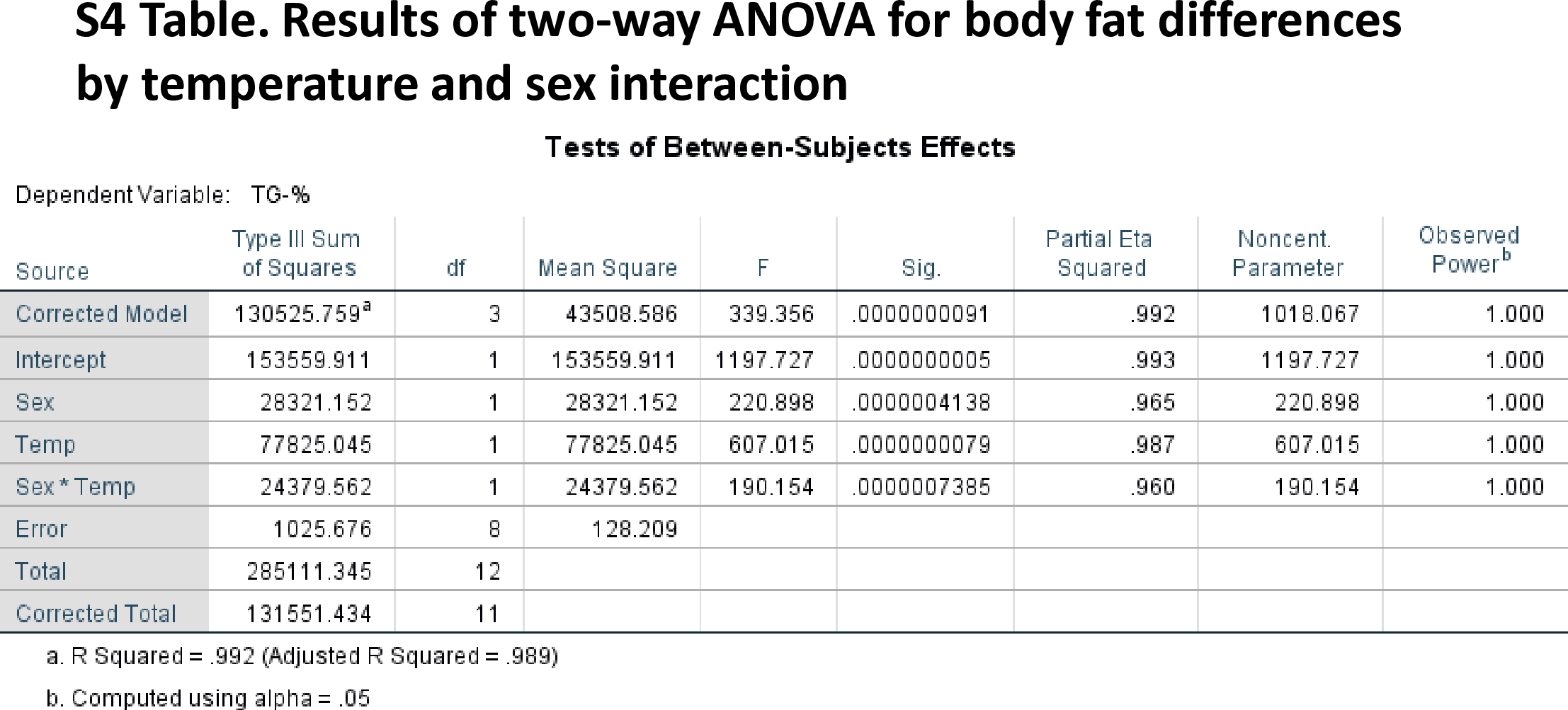

Supplement: S4 Table — (TIF) [file pone.0317971.s006.tif]

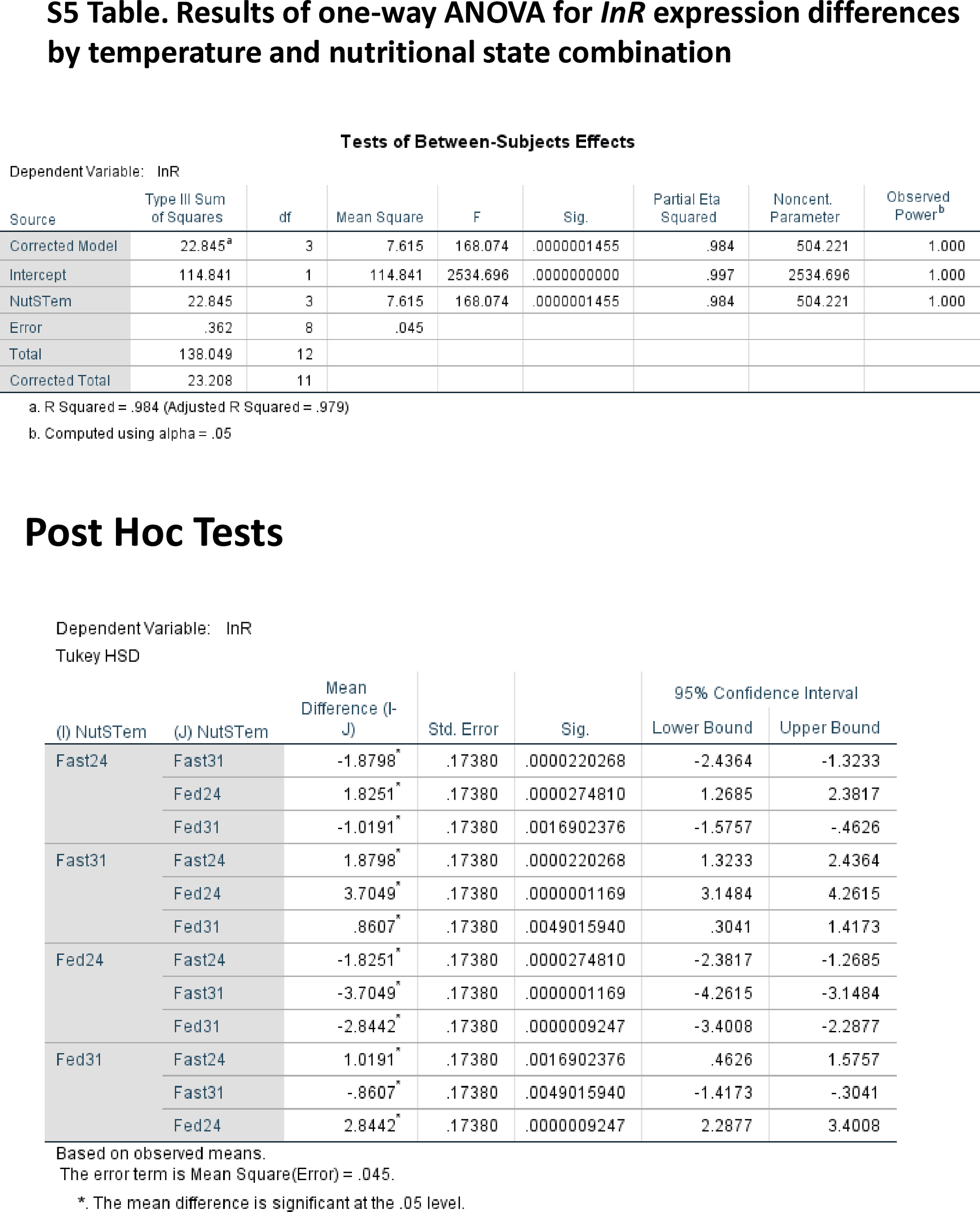

Supplement: S5 Table — Statistical differences among the four temperature and nutritional state combination groups were evaluated using a one-way ANOVA, followed by Tukey’s HSD post hoc tests (SPSS, version 26). The groups were Fast24 (Fasted-24°C), Fast31 (Fasted-31°C), Fed24 (Fed-24°C), and Fed31 (Fed-31°C). (TIF) [file pone.0317971.s007.tif]

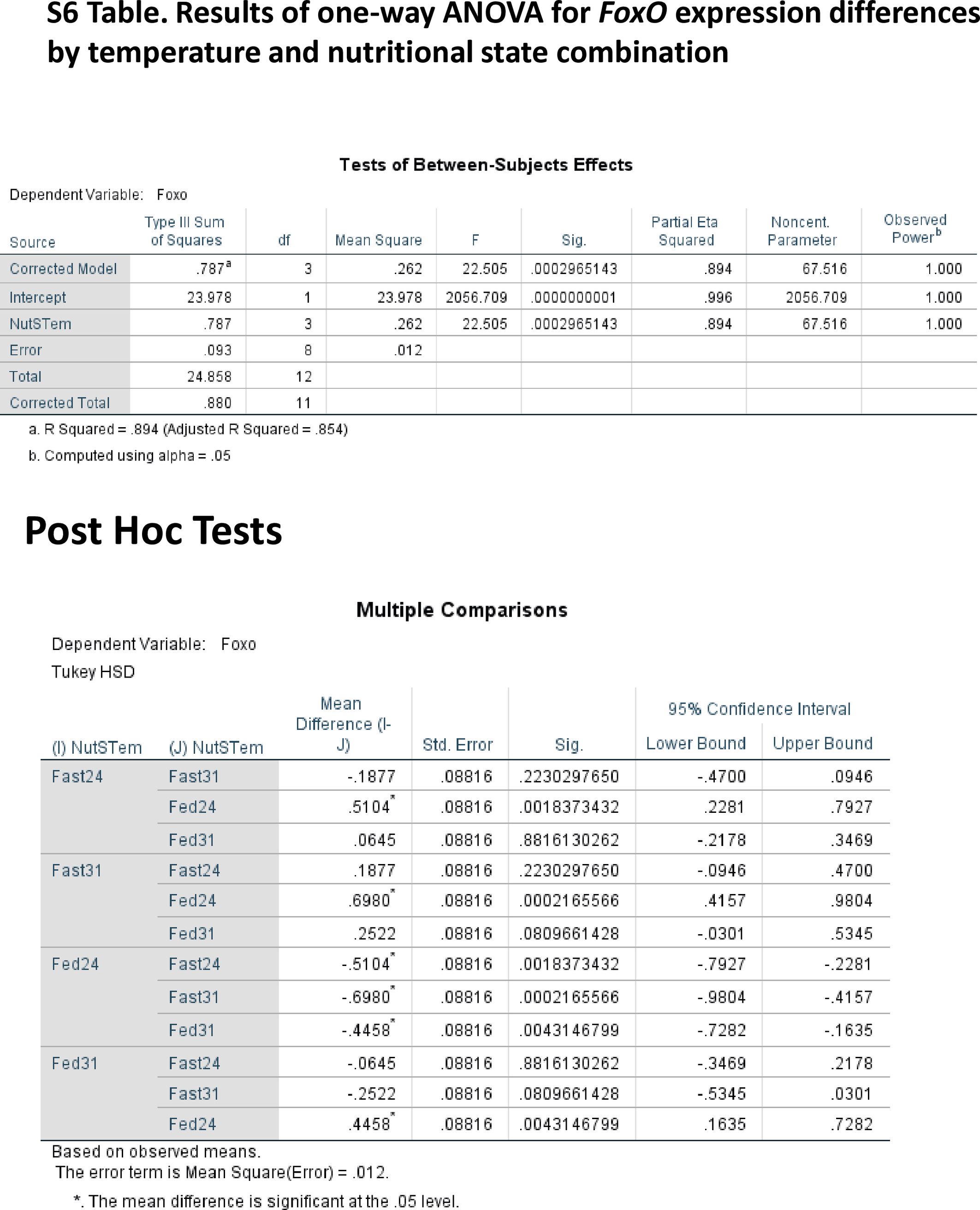

Supplement: S6 Table — Statistical differences among the four temperature and nutritional stage combination groups were evaluated using a one-way ANOVA, followed by Tukey’s HSD post hoc tests (SPSS, version 26). The groups were Fast24 (Fasted-24°C), Fast31 (Fasted-31°C), Fed24 (Fed-24°C), and Fed31 (Fed-31°C). (TIF) [file pone.0317971.s008.tif]

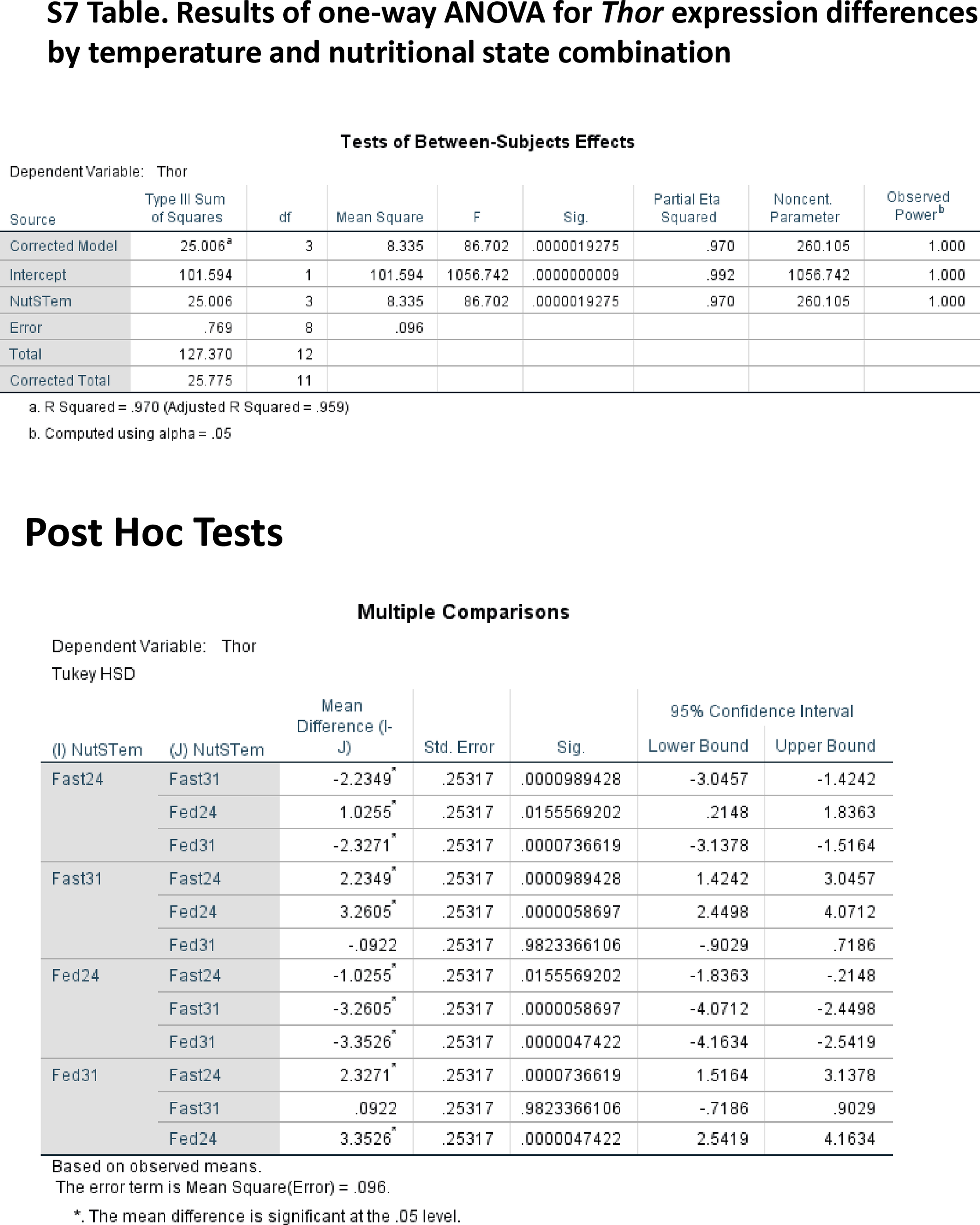

Supplement: S7 Table — Statistical differences among the four temperature and nutritional stage combination groups were evaluated using a one-way ANOVA, followed by Tukey’s HSD post hoc tests (SPSS, version 26). The groups were Fast24 (Fasted-24°C), Fast31 (Fasted-31°C), Fed24 (Fed-24°C), and Fed31 (Fed-31°C). (TIF) [file pone.0317971.s009.tif]

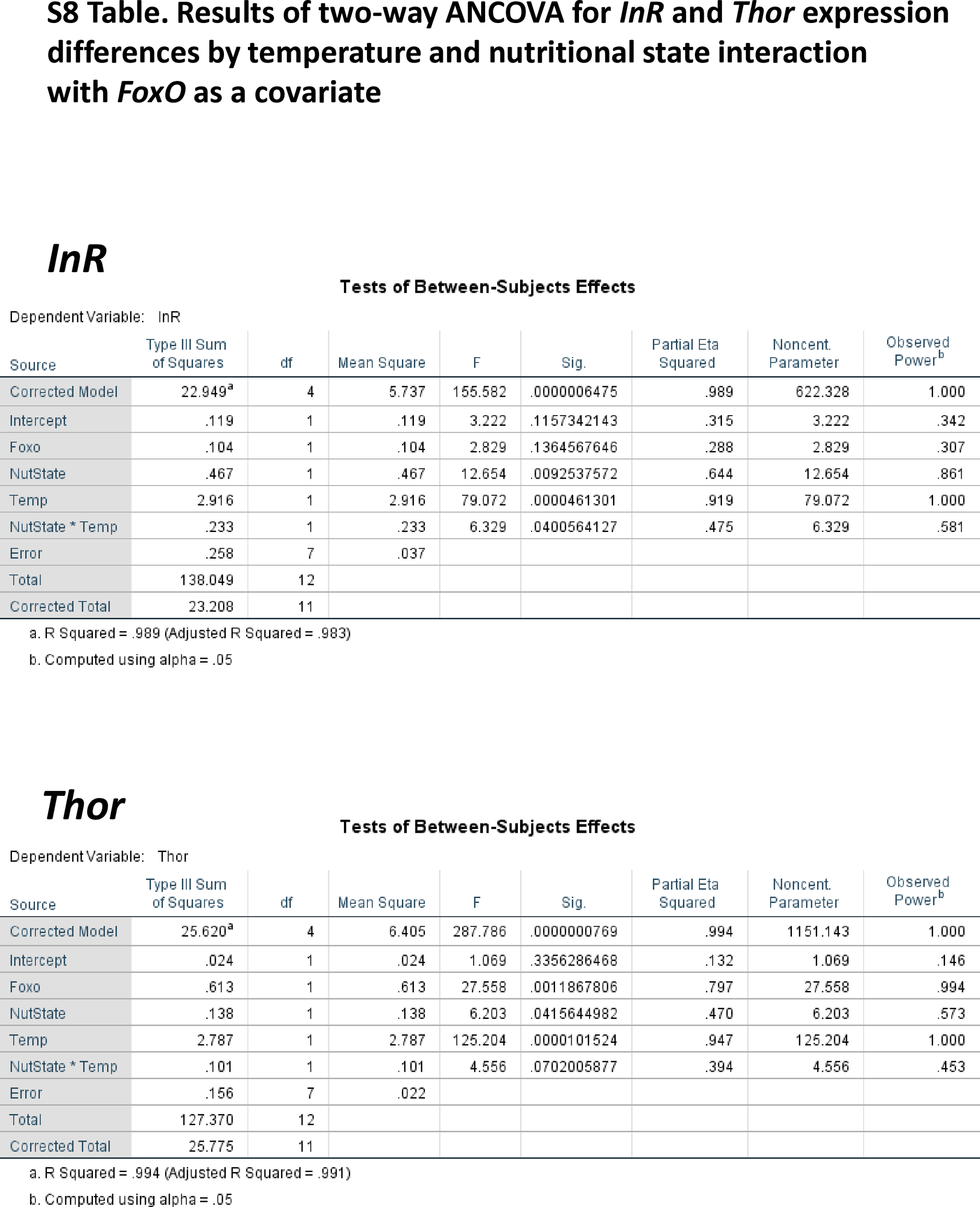

Supplement: S8 Table — (TIF) [file pone.0317971.s010.tif]

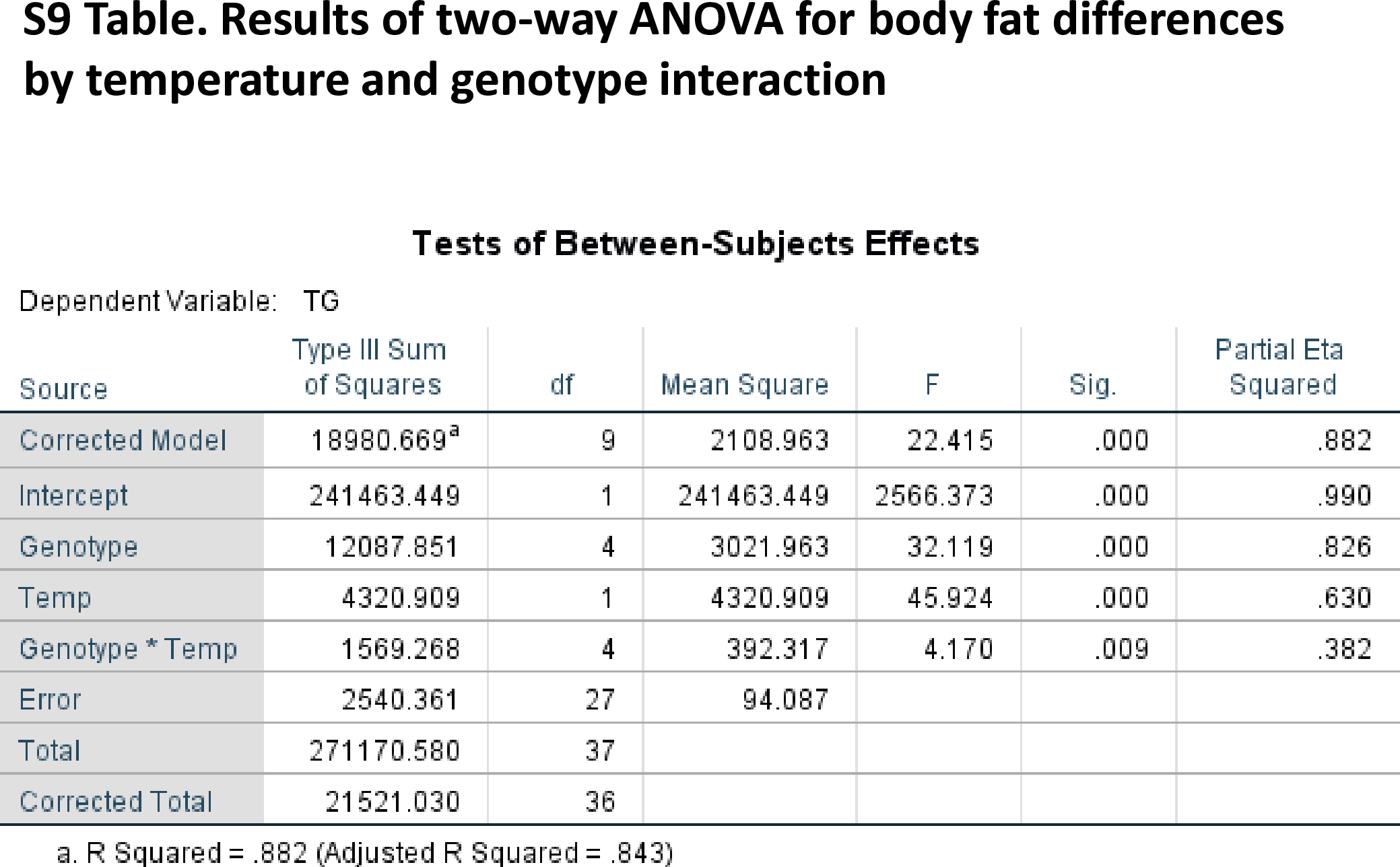

Supplement: S9 Table — (TIF) [file pone.0317971.s011.tif]

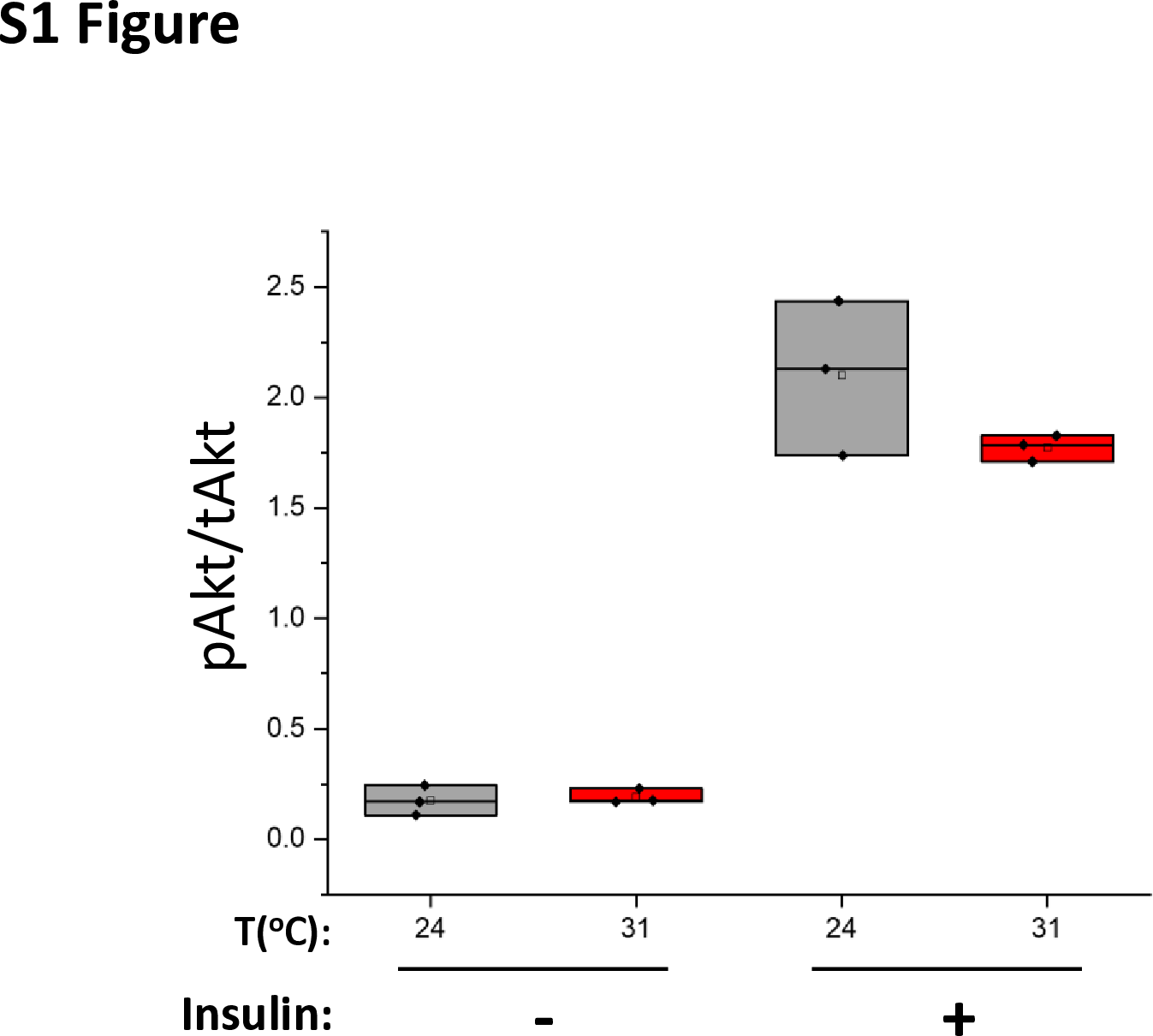

Supplement: S1 Fig — S2R+ cells were plated in 6-well plates and preincubated in nutrient-free media (NF-M) at 24°C or 31°C for 1 hour. Half of the wells remained in NF-M, while the other half were incubated with NF-M supplemented with 10 μg/ml insulin for an additional hour at their respective preincubated temperatures. Total protein extracts from S2R+ cells were prepared and analyzed by Western blot using antibodies specific to phosphorylated Akt (p-Akt) and total Akt (t-Akt). Images from three replicates (Fig 5 raw images) were analyzed using imageJ software to measure the densities of both p-Akt and t-Akt bands. Open squares represent the mean of pAkt/tAkt values, lines indicate the median, and individual data points are shown as dots. (TIF) [file pone.0317971.s012.tif]

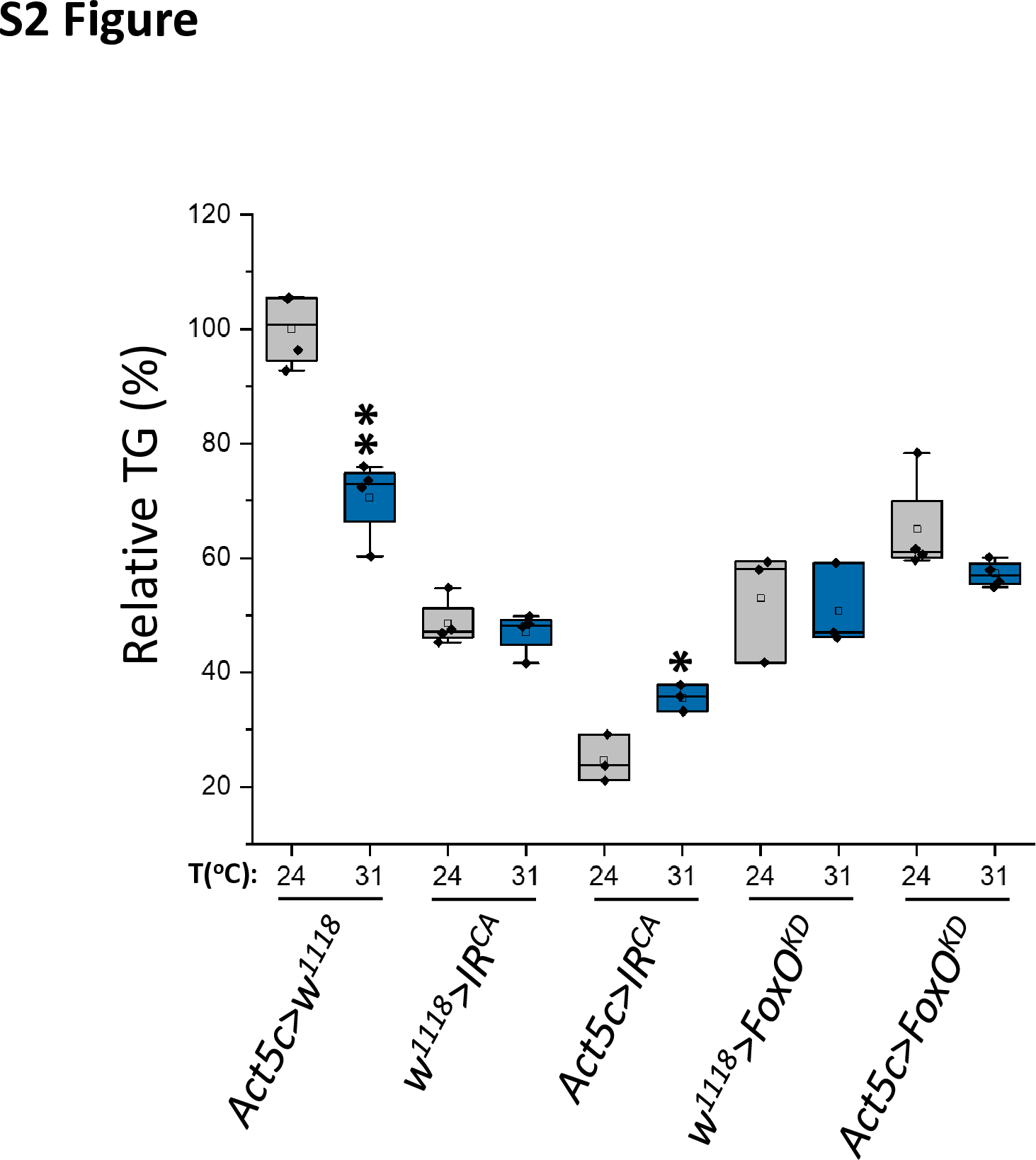

Supplement: S2 Fig — One-week-old male flies expressing a constitutively active form of the insulin receptor (Act5c > IRCA) and FoxO RNAi (Act5c > FoxOKD), along with control flies, were incubated at 24°C and 31°C for seven days. Triglyceride (TG) levels were then determined using a colorimetric method and normalized to the number of flies. The TG content of each experimental group was compared to that of control flies (Act5c > w1118), which was set to 100%. The relative amount of body fat (%) was calculated using the following formula. Relative Body Fat (%) = (TG: Experimental group/TG: Act5c > w1118, female at 24°C) X 100; Open squares represent the mean, lines indicate the median, and individual data points are shown as dots. Statistical differences among the two temperature groups in each genotype were evaluated using t-test. * p < 0.05, ** p < 0.01. Three or four replicates per group, six flies in each replicate. (TIF) [file pone.0317971.s013.tif]
